# Smart Bionic Surfaces with Switchable Wettability and Applications

**DOI:** 10.1007/s42235-021-0038-7

**Published:** 2021-06-11

**Authors:** Shuyi Li, Yuyan Fan, Yan Liu, Shichao Niu, Zhiwu Han, Luquan Ren

**Affiliations:** Key Laboratory of Bionic Engineering (Ministry of Education), Jilin University, Changchun, 130022 China

**Keywords:** bionic surfaces, external stimuli, switchable wettability, responsive mechanisms

## Abstract

In order to satisfy the needs of different applications and more complex intelligent devices, smart control of surface wettability will be necessary and desirable, which gradually become a hot spot and focus in the field of interface wetting. Herein, we review interfacial wetting states related to switchable wettability on superwettable materials, including several classical wetting models and liquid adhesive behaviors based on the surface of natural creatures with special wettability. This review mainly focuses on the recent developments of the smart surfaces with switchable wettability and the corresponding regulatory mechanisms under external stimuli, which is mainly governed by the transformation of surface chemical composition and geometrical structures. Among that, various external stimuli such as physical stimulation (temperature, light, electric, magnetic, mechanical stress), chemical stimulation (pH, ion, solvent) and dual or multi-triggered stimulation have been sought out to realize the regulation of surface wettability. Moreover, we also summarize the applications of smart surfaces in different fields, such as oil/water separation, programmable transportation, anti-biofouling, detection and delivery, smart soft robotic *etc.* Furthermore, current limitations and future perspective in the development of smart wetting surfaces are also given. This review aims to offer deep insights into the recent developments and responsive mechanisms in smart biomimetic surfaces with switchable wettability under external various stimuli, so as to provide a guidance for the design of smart surfaces and expand the scope of both fundamental research and practical applications.

## 1 Introduction

Wettability is essential for many biological processes, tremendous engineering and industrial technologies[1–6]. The organism's systems have evolved for billions of years to develop strategies for impeccable structure-property-performance relations, which will provide excellent blueprints for bionic designs. Especially, lots of natural surfaces with special wettability have attracted the attention of numerous scientific researchers, such as lotus leaves, rose petals, slippery pitcher plants, mosquito compound eyes, rice leaf, water striders and so forth, exhibiting such excellent functions as self-cleaning, anti-fouling and drag reduction *etc*[7–15]. According to the investigations of underlying mechanism of these natural objects, numerous researches on constructing biomimetic super-antiwetting surfaces are mainly governed by the cooperation of chemical composition with low surface energy and surface microstructures, which are widely used in both fundamental research and practical applications[16–18].

Up till now, researchers have developed various methods to construct artificial superwettable surfaces on different substrate, such as self-assembly, deposition, electro-spun, chemical-etching and so on[19–22]. However, most of studies on the surface wettability always are concentrated in single superhydrophobicity or superhydrophilicity. Recently, related researches on smart surfaces with dynamically switchable wettability in response to changes in surrounding environment have arose extensive attention on account of the wider range of industrial applications, such as the micro-fluidic devices, on-demanded oil/water separation, self-cleaning surfaces, lap-on-chip system, tunable optical lenses, and so forth[23–26]. Various external stimuli mainly including physical, chemical and dual or multi coupled factors can trigger the reversible wettability. Similar as the fabrication of superwetting surfaces, the transformation of surface chemical composition and geometrical structures fundamentally determines the switchable characteristics of wettability. Meanwhile, a variety of inorganic metal oxides and organic compounds usually are used as

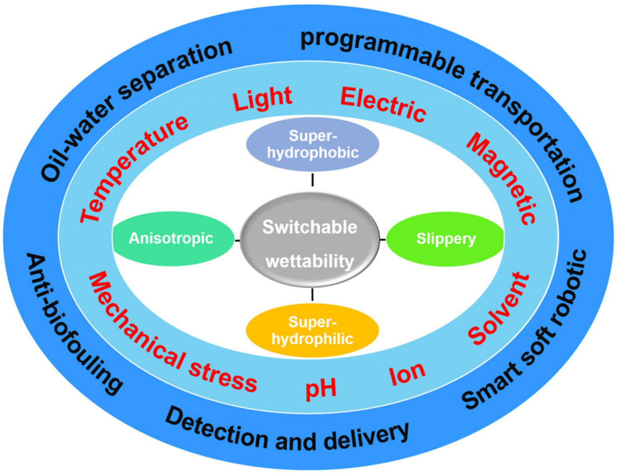

**Fig. 1** Diagram of the categories of switchable wettability, including superhydrophobic, superhydrophilic, anisotropic and slippery. External stimuli consisting of physical stimulation (temperature, light, electric, magnetic, mechanical stress), chemical stimulation (pH, ion, solvent) and dual or multi triggered stimulation will reduce the wettability transformation by changing the microstructures and chemical composition. All these studies can be applied in the field of oil/water separation, programmable transportation, anti-biofouling, detection and delivery, smart soft robotic.

stimuli-responsive substance[27–29]. By comparison, the organic polymers have advantages of not only more sensitive types (such as light, pH, thermal), conformational transitions (trans-cis), but also being able to simultaneously control the surface energy and topography. However, due to the structural and photochemical stability of inorganic oxides, it is difficult to achieve a change in morphology by responding to the external stimuli. Whereas, the use of inorganic oxides is low toxic and more environmentally friendly with a larger range of wettability changes. Thus, there is an urgent need for new design principles and engineering strategies to develop artificial surfaces with adjustable superwettability and special adhesion to solve smart materials with characteristics similar to natural examples.

This review aims to provide a summary of the recent developments in smart surfaces with switchable wettability (Fig. 1), composed of the following sections. Firstly, several special superwetting states (superhydrophobicity, superhydrophilicity, underwater superoleophobicity, super-lubricity, and different adhesive property) based on the natural creatures are used to illustrate the structural features and wetting mechanisms related to the switchable wettability. Secondly, this review focuses on the recent progress of the smart surfaces with switchable wettability and the corresponding regulatory mechanisms under external stimuli, which mainly is governed by the transformation of surface chemical composition and geometrical structures. Meanwhile, the external factors and mechanism affecting interfacial wettability have been introduced in detail, consisting of physical stimulation (temperature, light, electric, magnetic, mechanical stress), chemical stimulation (pH, ion, solvent) and dual or multi triggered stimulation. Thirdly, our attentions have been paid to the applications and the correlative changes of surface chemical components / microstructures, as well as several characteristics of oil/water separation, programmable transportation, anti-biofouling, detection and delivery, smart soft robotic *etc*. Finally, we briefly summarize the challenges in the development of smart wetting surfaces at present and the development tendency in future.

## 2 Mechanisms of interfacial superwettability on surfaces

Research on wettability can be traced back to more than two decades ago, the classical liquid wetting theoretical models usually include the Young's model on smooth solid surface (1805)[30], Wenzel's model on rough surface (1936)[31], and Cassie-Baxter model on inhomogeneous surface (1944)[32]. Up to date, based on the early theories, numerous researchers gradually began to describe lots of different types of special wettability on the surfaces of plants or animals in nature. Especially, special superwetting states have been reported in the past decade, including superhydrophobicity, superhydrophilicity, underwater superoleophobicity, super-lubricity *etc*. Meanwhile, the superwetting surface not only requires the contact angle to be greater than 150˚ for water or oil, but also reflects the hysteresis of the contact angle. Combined with the wettability property of typical creatures, the corresponding several special superwetting states shown in Fig. 2 will be expounded as follows.

The lotus leaf is considered a typical representative of superhydrophobic surfaces. There is an "air cushion" at the groove of micro-nanostructures to effectively reduce the contact area between water droplets and the

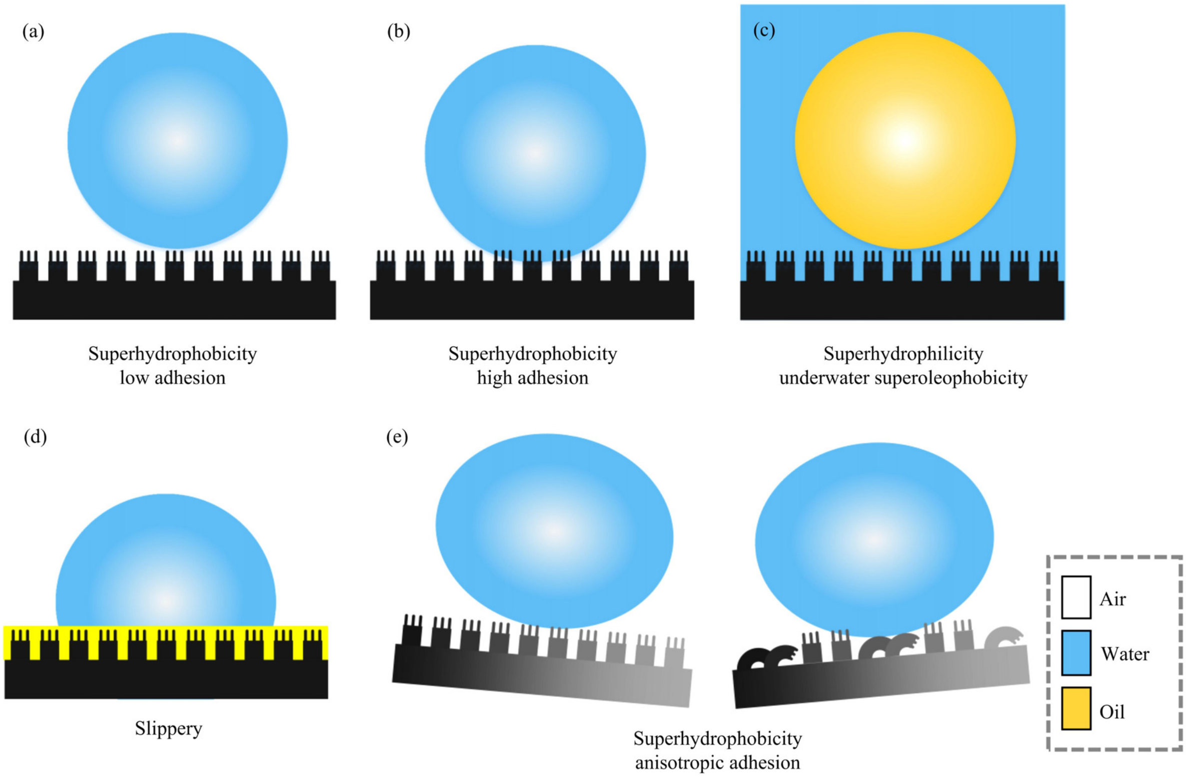

**Fig. 2** The illustration of different interfacial wetting states.

surfaces, thereby significantly inhibiting the lotus leaf from being wetted effectively and exhibiting super-hydrophobic self-cleaning phenomenon, which represents the special Cassie-Baxter state with ultra-low adhesion (Fig. 2a). Differently from the lotus effect, the surface of rose petals exhibits super-hydrophobic properties, but water droplets cannot move and be fixed on the surface. The small grooves among the micropillars are occupied with water and the large grooves among the nanofolds are forming an “air cushion” to maintain dry, which represents the wettability of Cassie impregnation state with high adhesion (Fig. 2b)[33]. Compared to the above water/air/solid interface, superhydrophilic fish scales surface exhibit underwater superoleophobicity because of the presence of water layer within the surface micro- and nanostructures instead of an air cushion, which can reduce the contact area between oil droplets and surface, thus preventing the adhesion of oil pollution[34] (Fig. 2c). Along with the extension of wettability research, introducing another immiscible liquid layer into solid surface microstructures also can realize the liquid repellency (Fig. 2d). Nepenthes pitcher plants with the Slippery Liquid-Infused Porous Surfaces (SLIPS), are difficult for insects to stop in the rim area and often “falling off” and slipping into the insect trap to be preyed[35]. Combined with the microtextural roughness, the surface of Nepenthes pitcher plant forms a highly stable lubricating layer, filling all the spaces in the texture, which is similar like the water layer on the fish scales. This feature allows it to repel and easily remove various liquids in the air[10]. Last but not least, compared with the isotropic rolling behavior of water droplets on the lotus leaf, the water droplets placed on the surface of the rice leaf and butterfly wings tend to fall along the direction parallel to the edge, exhibiting anisotropic superhydrophobicity, which resulting from the longitudinal grooves with a transverse sinusoidal pattern on rice leaves and aligned shingle-like scales on butterfly wings[8,36]. Unlike with the previous wetting state, the surface wettability presents the water droplets with a Cassie-like state and to form an extremely discontinuous Three-phase Contact Line (TCL) or a Wenzel-like state and to form a quasi-continuous TCL, respectively (Fig. 2e). The longitudinal grooves and scales on the rice leaves and butterfly wings effectively guide the droplets to roll directionally, resulting in the self-cleaning effect and inhibiting dust accumulation on the surfaces[37]. In the next section, this article will describe how to achieve intelligent switching between these wetting states by combining stimulus-responsive materials with microstructures or nanostructures, including the control of static wetting and dynamic wetting state.

## 3 Stimuli-responsive on switchable wettability

The wettability mechanisms of the above creatures provide an excellent reference for the design of superwetting surface, which will be useful for the explanation of relevant applications *via* the switchable wettability[38–40]. Smart switchable wettability of surface systems is reversibly triggered under various external stimuli, mainly including physical-responsive (temperature, light, electric field, magnetic field, stress, *etc*.) and chemical-responsive (pH, ion, solvents, *etc*.). Moreover, either single or dual or even multiple stimuli may work on a targeted property at the same time. This section will report and discuss various responsive mechanisms on the smart switchable wettability surface under different stimuli.

### 3.1 Physical stimulation

#### 3.1.1 Temperature

The temperature-responsive substance without dependent on other chemical additives, can achieve a fast and intense reaction process to control the wettability transformation, exhibiting significant advantages compared to many other environmentally stimuli[41–43]. Large numbers of thermo-responsive surfaces with switchable wettability have been developed in the past few years *via* grafting temperature-sensitive polymers, such as poly(N-isopropylacrylamide) (PNIPAAm), poly(2-isopropyl-2-oxazoline), poly(vinyl methyl ether), and poly(2-(dimethylamino)-ethyl methacrylate (PDMAEMA) *etc*., which exhibit state conversion in higher or lower temperature than their Lower Critical Solution Temperature (LCST) respectively[44–46]. For instance, PNIPAAm with a LCST about 32 ˚C – 33 ˚C is one of the utmost studied and widely used polymers for controlling the surface wettability[47,48]. Analyzing the thermo-driven wettability mechanism of the PNIPAAm, it is mainly due to the cooperation of intramolecular and intermolecular hydrogen bonding contributed by PNIPAAm chain. While the temperature is lower than the LCST, the surface is superhydrophilic, resulting from the effects of enthalpic contributions governed by comparing to the entropy contributions in this atmosphere, which is more likely to form intermolecular hydrogen between the N−H and C=O groups and water molecules. Oppositely, the entropy contributions will occupy the main position with a temperature above LCST, and the N−H and C=O groups preferentially form intramolecular hydrogen bonding, along with self-condensation and self-disintegration between the molecular chains to change into a collapsed conformation, repelling the water and exhibiting hydrophobicity. For example, Zhang *et al*. fabricated a thermo-responsive PNIPAAm-modified nylon membrane *via* hydrothermal method[49]. As shown in Fig. 3, by changing the temperatures lower or higher than the LCST, the switchable wettability of membrane exhibited the hydrophilicity and underwater superoleophobicity or hydrophobicity and superoleophilicity, so as to separate various stabilized oil-water emulsions effectively.

Especially, dynamic regulation of wettability reversibly also can be achieved by designing a special liquid, which can react with the functional surface in

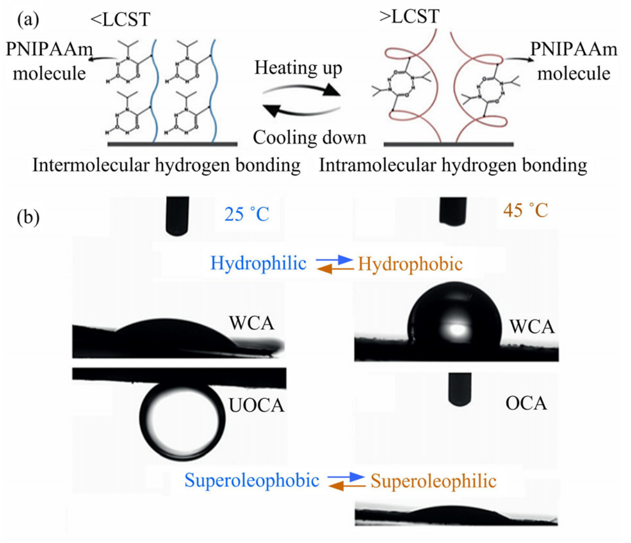

**Fig. 3** (a) The responsive mechanism of the thermo-driven PNIPAAm; (b) the reversible wettability between the hydrophoilic/superoleophobic and hydrophobic/superoleophilic at 25 ˚C and 45 ˚C[49].

response to temperature. For example, by grafting the mixed molecular brushes of poly(phenylethyl methacrylate) and 1H,1H,2H,2H-perfluorodecyltrimethoxysilane (PPhEtMA-co-PFDMS) on the surface, Chang *et al.* first fabricated a thermo-induced surface, showing switchable wettability by an ion liquid (1-ethyl-3-methyl imidazolium bis(trifluoromethylsulfonyl)imide ([EMIm][NTf2])[46]. The dynamical regulation of surface wettability was due to the changes of cation–π interaction between [EMIm][NTf2] and PPhEtMA-co-PFDMS brushes under different temperature in Fig. 4a. Similarly, the conversion of dynamic surface wettability can also be achieved. Wang *et al.* directly designed a biological droplet adhesion system containing single-stranded DNA with reversible molecular configuration deformation under thermal stimuli, realizing the precisely controlment on the n-dodecane-infused slippery surface[50]. As shown in Fig. 4b, while increasing the temperature from 283 K to 303 K, the flexibility and mobility of ssDNA were improved, along with the transformation of molecular conformation with the exposure limitation of hydrophobic moiety, which limit the interaction between the hydrophobic group and the lubricating molecule, thereby reducing interfacial adhesion.

In addition, several works on reversible superwetting transition were achieved by the heating treatment and remodification[51,52]. Notably, combined the removal of n-dodecanethiol (hydrophobic materials) at 350 ˚C and remodified at room temperature, Zhang *et al.* designed a biomimetic switchable wetting surface on AZ91D Mg alloy inspired by Lotus Seedpod[53]. Especially, different from the examples about the thermo-induced wettability mechanism mentioned above, Zhang *et al.* fabricated a superhydrophobic and superoleophilic nanoparticle film with reversible wettability *via* spray deposition, in which wetting transition from Cassie to Wenzel model is due to the water vapor condensation on the surface by adjusting the temperature[54].

### 3.1.2 Light

As well known, the controlment of light has the characteristics of rapid contact, high resolution and remote controlment *etc*., so triggering changes in surface wettability through light-response has attracted widespread attention. The active molecules on the light-responsive material surface will occur reversible changes in chemical composition, chemical configuration or polarity *etc*., which can cause a change in the surface free energy, resulting in the reversible changes in

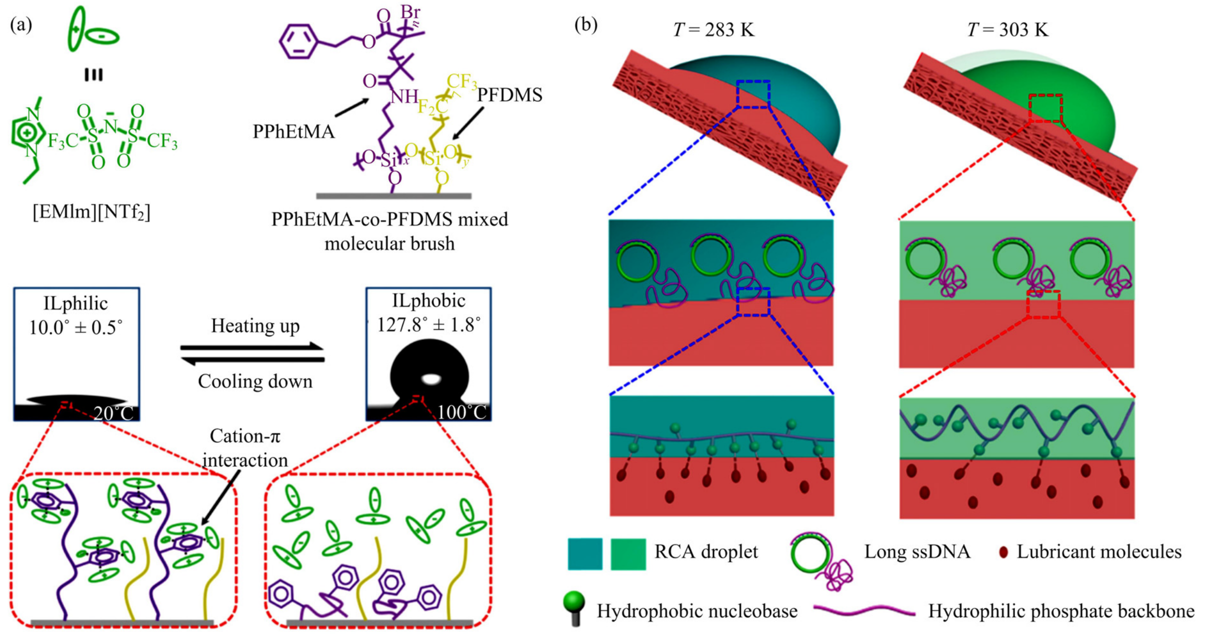

**Fig. 4** (a) The dynamic regulation of temperature-responsive wettability by an ion liquid ([EMIm][NTf2])[46]; (b) schematic illustration of the hydrophobic interaction at the interface of biological droplet and n-dodecane under different temperature[50].

wettability.

The inorganic semiconductor oxide materials with optical triggering-yielded electron-hole pairs usually are used to regulate the reversible light-responsive wettability, such as titanium dioxide ($TiO_2$), zinc oxide (ZnO), tin dioxide ($SnO_2$), tungsten trioxide ($WO_3$) and vanadium oxide ($V_2O_5$) *etc*[29,55,56]. In the preparation of wettability-regulating superwettability surfaces, photosensitive inorganic semiconductor materials mainly utilize the free conversion of oxygen vacancies and hydroxyl groups under ultraviolet (UV) light irradiation and dark treatment (or heat treatment) to change the surface chemical polarity, thereby achieving the switchable wettability. The preparation method comprises the steps of preparing directly on the photosensitive material substrate, or grafting the photosensitive metal material nanoparticles on different substrates by a certain process[57,58]. Taken $TiO_2$ as an example, the wetting transformation mechanism and the fabrication methods are explained briefly as follows. The electrons of $TiO_2$ will be excited from the valence band to the conduction band under UV irradiation, and the photo-induced electron-hole pairs are generated, leading to the cleavage of Ti−O bond, resulting in the formation of oxygen vacancies, thereby enhancing the adsorption of hydroxyl groups and some coexisting molecular water, so as to exhibit a superhydrophilic state (Fig. 5a)[29]. During heating process, the hydroxy groups are replaced by oxygen atoms that have a stronger bond on the defect sites, restoring the initial superhydrophobic properties. Kang *et al*. prepared $TiO_2$ nanotube arrays on the Ti sheets *via* a simple anodizing and heating process in Fig 5b. Reversible transition between under-oil superhydrophilicity and under-oil superhydrophobicity can be realized by alternation of UV irradiation and heating treatment. Yong *et al*. fabricated regular micro/nanoscale hierarchical rough $TiO_2$ structures on Ti substrate by femtosecond laser ablation, realizing the switchable underwater superoleophobicity-superoleophilicity by alternating UV irradiation and dark storage for the first time (Fig. 5c)[59]. Differently, as shown in Fig. 5d, *via* ultrasonic assisted dip coating, Li *et al*. fabricated an intelligent and robust sponge by grafting $TiO_2$ nanoparticles and octadecanoic acid, achieving the properties of smart switchable super-wettability and effective oil-water separation under UV illumination and heating[60].

Except for inorganic compounds, several organic molecules also possess the characteristic of photo-responsive wettability with a shorter time to respond

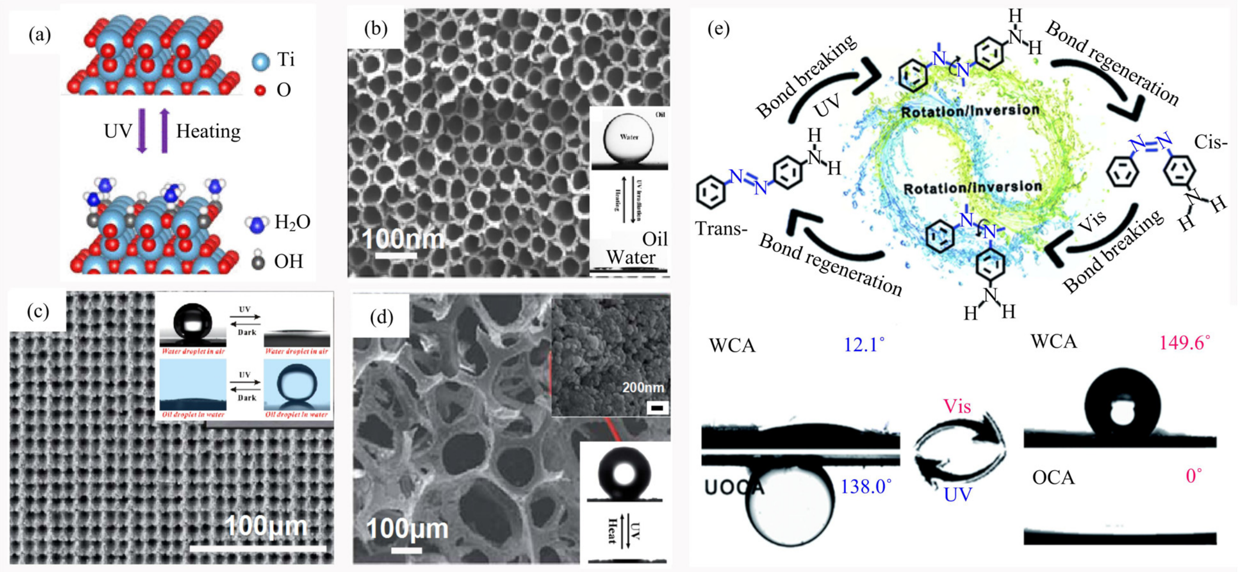

**Fig. 5** (a) Schematic diagram of the anatase $TiO_2$ (004) facet crystal structure variation under UV irradiation and heating, applying for the explanation of the light-induced transformation mechanism of surface wettability[29]. SEM images of $TiO_2$ nanotube arrays fabricated by anodizing and heating (b)[29], femtosecond laser-ablated Ti surface (c)[59], sponge modified with $TiO_2$ nanoparticles (d) and the reversible wettability under UV irradiation and dark environment or heat treatment[69]. (e) The conformational conversion mechanism of photo-responsive organic molecules and wettability transformation of WCA/OCA changes on the modified copper mesh under UV and Vis light[61].

and recover, due to the photoisomerization-induced reversible conformational conversion and/or the dipole moment change, as well as spiropyran and azobenzene *etc*[62–65]. For example, azobenzene compounds with excellent trans-cis isomerism properties (shown in Fig. 5e), are easy to cause the transition of surface polarity under the irradiation of UV and visible light, thereby resulting in the regulation of surface wettability[27,61,66,67]. By means of modifying the nano-Ag pine needles and aminoazobenzene (AABN) on polydopamine (PDA) pre-treated porous meshes, functional surfaces with reversible photo-responsive wettability from highly hydrophobic to superhydrophilic were fabricated by Qu *et al.*[61]. The wettability transformation was ascribed to the alternate exposure of the hydrophilic group (Ag) and hydrophobic benzene ring from AABN. Under UV irradiation, the N=N bond of AABN was broken and rotated, resulting in the isomerization conversion from trans-form with molecule stood up straight to cis-form with molecule lying down, and exposing the hydrophilic Ag pine nano-needles. While irradiated by visible light, the molecule conformation was transformed into trans-form, and the initial highly hydrophobicity recovered again, revealing a larger transformation extent in reversible wettability.

### 3.1.3 Electric

Comparatively speaking, the stimuli of electric field possess the property of super-fast responsiveness and convenient controlment, causing the changes in the interfacial energy, which has powerful potential in the smart transition of surface wettability[68,69].

One method is to modulate the droplet behavior through the electric-induced local molecular reorganization. Recently, lots of electric-responsive surface based on conducting polymer have been used to realize the switchable wettability, such as polythiophene (PT), polypyrrole (PPy), and polyaniline (PANI) *etc*[70–72]. *Via* the self-assembling of (16-Mercapto) hexadecanoic acid (MHA) onto the Au (111), Lahann *et al.* designed a surface with dynamically interfacial wettability in response to electric potential, which was caused by conformational transitions between hydrophilic and moderately hydrophobic state (Fig. 6a)[73]. Combined colloidal lithography with electrodeposited polythiophene derivatives, Pernites *et al.* fabricated a superhydrophobic polythiophene film with electric-responsive switchable wettability between hydrophilicity and superhydrophobicity by changing the redox property of conducting polymer, achieving the attachment or unattachment of both fibrinogen protein and *Escherichia coli* under the switching of on/off the low potential (1.05 V) (Fig 6b)[70]. Instead of the conducting polymer, changing the surface energy of conductive inorganic materials also can achieve the transformation in wettability. By electrodepositing tin layer on the copper electrode, Wang *et al.* obtained a copper/tin system with in situ reversible superwetting transition between underwater superhydrophilic and superoleophobic properties by electrochemical atomic alternation (Fig. 6c)[74]. The deposition of tin can significantly reduce the surface energy of the copper electrode and can be dissolved by removing the potential, thereby restoring the initial high energy state of the copper, which can realize the whole in situ reversible superwetting conversion via the switch of on/off potential.

The other route is directly applying the electrical field onto the liquid and conductive substrate to obtain the switchable wettability[75,76]. Krupenkin *et al.* demonstrated the dynamic electrical control over the wetting behavior on the nanostructured surface for the first time, transforming from superhydrophobic state to almost complete wetting under 22 V (Fig. 6d)[77]. The changes relied on the applied voltage and liquid surface tension, causing the liquid permeation in the nanostructure layer. In addition, Kavousanakis *et al.* demonstrated the reversible change of contact angle in response to an electric field was closely related with the thickness of the solid dielectric (Fig. 6e)[78].

### 3.1.4 Magnetic

The magnetic-responsive process is easy to operate, low energy consumption, safety and fast responsiveness etc. Magnetic field-driven reversible wettability can dynamically control surface features by embedding magnetic particles or magnetic fluids[79,80]. The magnetic-induced mechanism is mainly attributed to the changes in surface microstructures[81–86]. Combining spray coating with magnetic-field-directed self-assembly, Yang *et al.* fabricated a magnetic-responsive

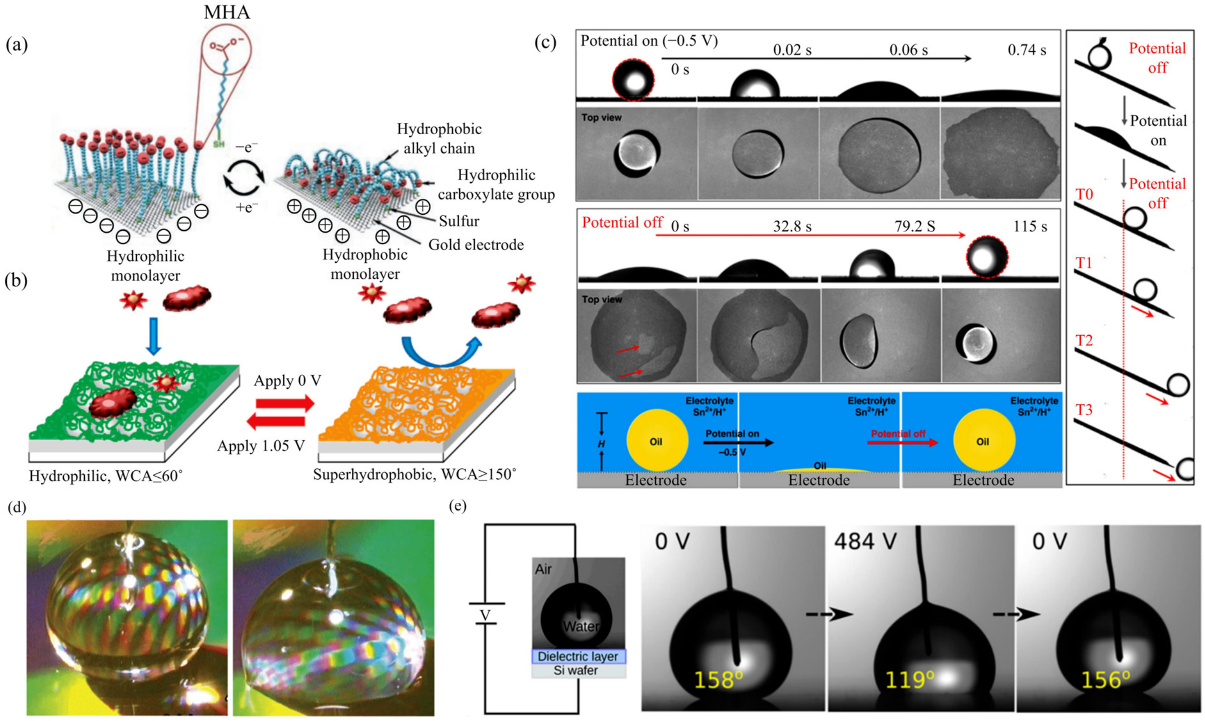

**Fig. 6** (a) The transition mechanism between straight (hydrophilic) and bent (hydrophobic) molecular conformations of MHA[73]. (b) Protein and bacterial adhesion and wettability onto the undoped (orange-colored film) and doped (green-colored film) colloidally templated polythiophene film[70]. (c) The *in situ* reversible superwetting transition between underwater superhydrophilic and superoleophobic properties by electrochemical atomic alternation[74]. (d) Appling the voltage between the droplet and the substrate, different wetting states on the nanostructured substrate[77]. (e) The contact angle reversibility under the on/off voltage of 484 V[78].

superhydrophobic surface with a dense array of magnetorheological elastomer micropillars (MREMPs)[87]. As shown in Fig. 7a, by tuning magnetic fields, the microstructures of MREMPs transformed from the collapsed state to the fully upright position, resulting in the surface adhesion changing from adhesive state to rolling state. Relying on the distribution of the magnetic nanoparticles inside the pillars, Drotlef *et al*. designed the arrays of actuated magnetic micropillars, which can be tilted, twisted, and rotated by varying the strength and the direction of the magnetic field gradient (Fig. 7b)[88]. At the same time, because the magnetically induced transformation of pattern geometry, the rolling angle of water droplets on the surface exhibited the magnetically direction-dependent wetting changes, which also can be used to obtain the transfer of microparticles. As shown in Fig. 7c, Huang *et al*. designed and fabricated a transformable surface consisting of an array of magnetic-responsive hierarchical micropillars[89]. By transforming the surface morphologies under an external magnetic field, the dynamically switchable wettability between the superhydrophobic and slippery states can be easily obtained.

### 3.1.5 Mechanical stress

Compared with the above stimuli, applying mechanical stress to achieve reversible controlment of surface wettability has the characteristics of faster, significant, continuous, and environmentally friendly[90]. Similarly, the regulation mechanism of surface wettability is the same as magnetic-induced transition, which is tuned by manipulating the microstructures.

Recently, as a kind of smart material, Shape Memory Polymers (SMPs) can recover their original shape after deformation by external stimulus[25]. On the basis of the shape memory effect of diglycidyl ether of bisphenol A (DGEBA) epoxy resin, Cheng's group demonstrated the intelligent regulation of epoxy SMP micro-nano array surfaces in rewritable microfluidic chips, anisotropic wettability, interfacial solid-liquid adhesion properties and repairable superhydrophobic surfaces[91–94]. As shown in Fig. 8a, under external force,

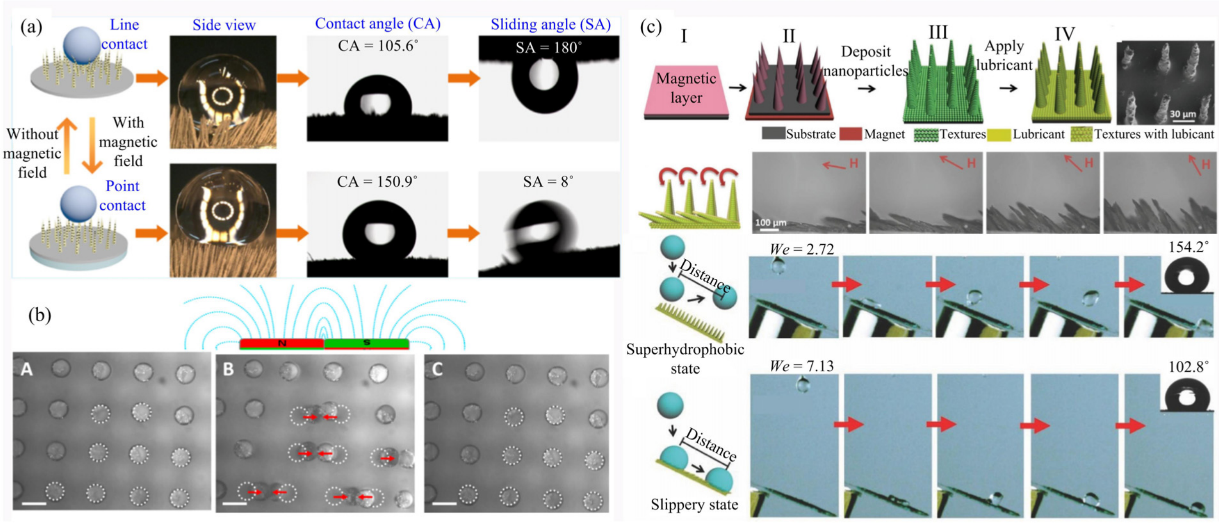

**Fig. 7** (a) Switchable wettability and adhesion induced by the morphology of MREMPs under the on/off switching of magnetic field[87]. (b) The movement of micropillars in opposite directions with a magnetic gradient[88]. (c) Surface transformation between superhydrophobic and slippery states under directional magnetic field[89].

the surface microstructures on the epoxy SMP collapsed, showing high adhesion. However, the heat treatment can make the surface microstructure and adhesion properties restore to initial state owing to the shape memory characteristics, thereby realizing the reversible conversion of water droplets between low adhesion and high adhesion on the surface. In addition, the transformation between the lotus-leaf-like random state and the rice-leaf-like 1D ordered state was also realized due to the presence/absence of the microgroove structures by alternating mechanical stress and heating treatment, resulting in the superhydrophobic switching between the isotropic and anisotropic wettings.

In addition, several flexible materials also can be used for the transformation of the wetting state through structural changes. As shown in Fig 8b, Wang *et al.* fabricated a smart skin-like PDMS elastomer surface with dynamic wetting behaviors by laser direct writing technology[95]. The surface topography can be finely regulated by tuning the direction and strength of the exerted force, leading to the superhydrophobic wettability switch quickly between the “lotus effect” and “rose effect”. Based on the transformation mechanism, they also applied the smart artificial skin to the joints of fingers, realizing the capture and release of water droplets only by finger motions without external energy supply or appliance. Similarly, by changing the surface microstructure under mechanical stress, Wu *et al.* designed an elastic regular array of pillars on PDMS substrate by using two-beam laser interference lithography and imprint lithography[96]. The adhesion force and sliding angle of treated PDMS substrate exhibited strong dependence on the surface curvature, which can achieve *in situ* switching wettability between pinned and roll-down superhydrophobic states, thus realizing the no-loss water droplet transportation.

## 3.2 Chemical stimulation

### 3.2.1 pH

As pH is one of the most common and easiest stimuli to regulate environmental response signals, pH-responsive substances nowadays have been utilized in various fields, such as drug delivery, separation and biosensors. Plenty of compounds, mainly containing carboxyl, pyridine and tertiary amine groups, can be used to respond acidic or alkali aqueous through protonation and deprotonation of functional groups, such as poly(acrylic acid) (PAA) and poly(methacrylic acid) (PMAA), poly(vinylpyridine) (PVP), poly(dimethylamino) ethyl methacrylate (PDMAEMA), and $HS(CH_2)_{10}COOH$ *etc*[97–102].

Pyridine, an acid induced swollen group, can be protonated under acidic aqueous, which is often used for the transformation of pH-responsive reversible wettability. By modifying the Porous Anodic Aluminum Oxide (PAAO) membrane with the pH-responsive

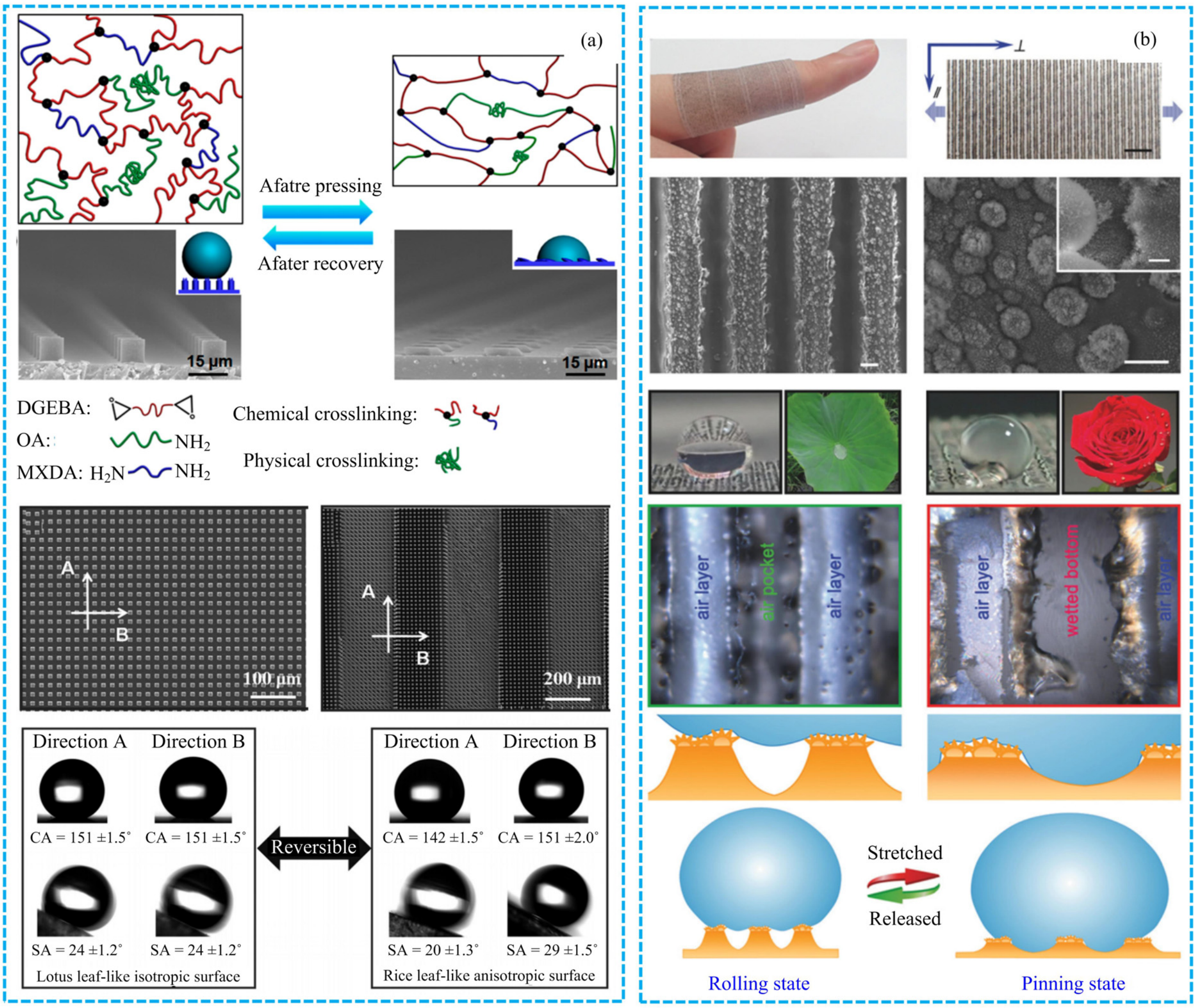

**Fig. 8** (a) Schematic illustration of molecular chain conformation and wetting models of SMPs with/without pressing[93]. Surface microstructure variation between the lotus-leaf-like and rice-leaf-like structure shapes and the corresponding wetting results, concluding the CA and SA. Directions A and B are the measured directions as labeled in images[94]. (b)Wearable structured surface with periodic gratings and different wetting state with or without stretching strain[95].

poly(4-vinylpyridine) (P4VP) and hydrophobic/oleophilic polystyrene block copolymer, Cai *et al.* fabricated a smart surface on a PAAO membrane with the characteristic of antifouling and pH-responsive oil wettability (Fig. 9a)[103]. While immersed in acidic water, P4VP chains protonated and exposed, resulting in the hydrophilicity and superoleophobicity. However, the membrane transformed into initial superoleophilicity quickly while move into the neutral water and drying. In contrast to the acid induced swollen pyridine group, carboxylic group, an alkali induced swollen group, possess the similar pH-induced wettability. While the pH is lower (or higher) than its pKa (around 5–6), the carboxyl groups will be protonated (or deprotonated), resulting in hydrophobicity (or hydrophilicity). Through grafting acrylic acid (AA) and acrylamide (AM) onto the eucalyptus pulp cellulose *via* Ce (IV)-initiated free-radical polymerization, Cheng *et al.* fabricated a smart pH-responsive surface with reversible wettability (Fig. 9b)[102]. Due to the presence of oppositely charged groups in terms of pH-responsivity, as the pH values switched between 1 and 9, the amino or carbonyl group on the compound surface undergone protonation or deprotonation, causing switchable wettability between oleophobic-hydrophilic and hydrophobic-oleophilic, so as to achieve the controlled oil-water separation.

Nevertheless, it can be found that pH-responsive copolymers often require relative longer time to achieve the wettability transition. In comparison with the ordinary

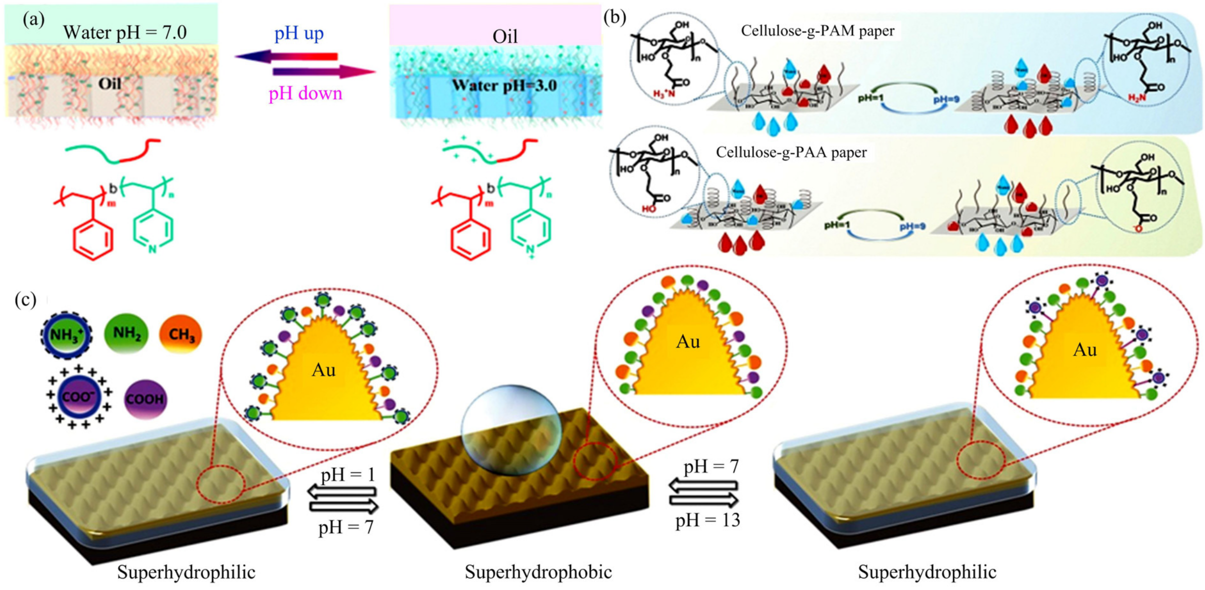

**Fig. 9** The pH-responsive mechanism of reversible wettability of the surface grafted with different compounds. (a) Acid-induced swollen pyridine group, schematic illustration of the switchable oil wettability of the PS-b-P4VP-grafted PAAO membrane under acidic aqueous[103]. (b) The carboxylic group in PAA and amino groups in PAM under different pH, the cellulose-g-PAA and cellulose-g-PAM paper with opposite switchable oil/water wettability[102]. (c) Illustration of the various surface modified by the mixed thiols under different pH conditions[99].

free radical polymerizations, modification with thiol molecules (such as $HS(CH_2)_{11}OH$, $HS(CH_2)_9CH_3$, $HS(CH_2)_{10}COOH$) is more likely to reduce the complicated synthesis process of smart separation materials (Fig. 9c)[99]. For example, combined the porous nylon membrane with self-assembled thiol molecules, Li *et al*. fabricated a smart membrane with tunable wettability property upon the pH variation, effectively separating various oil/water mixtures and surfactant-stabilized emulsions with excellent recyclability of the long-term separation[104]. Similarly, through the grafting of $HS(CH_2)_{11}CH_3$ and $HS(CH_2)_{10}COOH$, Liu *et al*. fabricated pH-responsive super-wettable surfaces on the metal substrate, such as copper foams, aluminum alloy and copper alloy[105–107]. The switchable surface wettability induced by pH-triggered protonation and deprotonation processes, endowed the as-prepared specimens with the properties of continuously separating the oil/water mixtures, or manipulating or storing types of microdroplets. Even though the pH-responsive surfaces with rapid wettability transformation is critical in potential applications, there are still several obstacles to be resolved, such as the pH sensitivity enhancement, large variation in wettability and eco-friendly *etc*., which limits its application to some extent.

### 3.2.2 Ion

Designing ion-responsive surfaces open up a new strategy to tune wettability dynamically, which is induced by ion-pairing interactions between cationic or anionic electrolytes and their complexes[108–110]. Based on the characteristic of reversible counterion exchange, series of materials consisting of amine or nitrogen heterocycles with a quaternized moiety, such as polypyrrole, quarterized amine polymers, ionic liquid-based polymers or quaternary ammonium thiolated molecules etc., are used for the control of wettability[111–113]. *Via* self-assembly method, Osicka *et al*.[114] fabricated rough-structured gold surface with Self-Assembled Monolayer (SAM) on a silicon wafer, consisting of positively charged quaternary ammonium group with aliphatic tail bearing terminal thiol functionality. This work achieved the switchable wettability from superhydrophilic and superhydrophobic state by counterion exchange from Cl− to perfluorooctanoate (PFO−) firstly. Combined with the kinetics of counterion exchange, the changes in wettability are ascribed for a long hydration process and strong electron ion pairing between quaternary ammonium group and perfluorooctanoate counterion. On account of the salt-responsive property of zwitterionic polymer brushes, Zheng's teamwork developed poly(3-(1-(4-vinylbenzyl)-1H-imidazol-3-ium-3-yl) propane-1-sulfonate) (poly VBIPS) to switch surface wettability from 40˚ to 25˚, while exposed to Phosphate Buffer Saline (PBS) in water or sodium chloride solution[115,116]. The salt-responsive behaviors can be explained by the effects of ionic strengths on the chain conformation of poly VBIPS brushes. While in the PBS/water, the polymers' chain conformation collapsed to realize surface adhesion, but with an extended state in NaCl solution to obtain antifouling property, thereby switching the protein capture/release reversibly in the biomedical application.

In addition, metal-ion-induced in dynamically wettability transition always have been reported. By grafting the liner poly(acrylic acid) (PAA) onto the polydopamine-coated stainless-steel mesh, Xu *et al.* developed a new method for achieving the wettability transition on basis of the chelation between $Hg^{2+}$ and PAA[117]. The as-prepared superhydrophilic mesh transformed into a highly hydrophobic with a contact value about 149˚ after immersed in $Hg^{2+}$ solution, which can be used as an ideal material for the $Hg^{2+}$ detection and oil-polluted water purification. *Via* one-step immersion in salt solution ($FeCl_3$), the N atoms in the intrinsically hydrophilic melamine sponge coordinated with the transition metal ions ($Fe^{3+}$), and a highly hydrophobic and oleophilic sponge was prepared by Ding *et al.*[118].

### 3.2.3 Solvent

The influence of the surrounding media on the wettability is mainly reflected in the sensitivity of the smart surface to the solvent[119–122]. Among the reported solvent-responsive polymers, the reason for the transition in switchable wettability is caused by the change of interfacial free energy owing to the configurational changes in polymer chains[123,124]. Liu *et al.* fabricated a Janus actuator with superhydrophobic and hydrophobic sides with the mixtures of PDMS and graphene *via* laser etching, realizing on-off switchable ability between an oil/water mixture and ethanol due to the changes of surface tension and absorption expansion[125]. Differently, by adding sugar or diluting to water, Yong *et al.* realized the no-loss oil droplet transportation on the femtosecond laser-irradiated silicon surfaces in a water environment[126]. When adding sugar, the density of water solution surrounding oil droplet increased, as well as the buoyancy acting on the oil droplet was larger than the gravity, causing an oil droplet to be picked up. While diluting the water, the oil droplet was put down. By changing the density of the solution (larger or lower than that of the oil), this surface is used to achieve the transport of oil droplets as an *in situ* "mechanical hand". However, compared with the above stimuli-responsive smart surfaces, most of the solvent-responsive surfaces with switchable wettability are often only sensitive to one or a few specific solvents, greatly limiting their applications.

Finally, in order to compare relevant studies parallelly, summative words for the above works are further listed in Table 1. It mainly includes the corresponding responsive materials used for the preparation of smart reversible surfaces, the achieved wetting performances, features in surface control, the advantages and disadvantages of surface wetting control.

## 3.3 Dual/Multi-stimuli

Recently, dual and multiple responsive surfaces with switchable wettability have been developed to respond to multiple triggers simultaneously or independently. This will provide a more effective strategy for functional control from different directions and satisfy basic research and industrial applications[127–131]. Based on as-prepared hydrogels in response to stretch, pH and temperature, Huang *et al.*[132] built a system with controllable wettability responding to multiple types of stimuli simultaneously (Fig. 10a). The wettability transition was consistent with the hydrogel state in constriction or expansion. And that, a container made by different composite materials contained a dye of different color, was used to directly demonstrate the specific changes in wettability. Beyond that, combined the tunable surface microstructure owing to the shape memory effect of SMPs and the temperature-responsive PNIPAAm with switchable hydrophilicity/hydrophobicity, Zhang *et al.*[133] also designed various smart gradient wetting surface with responsivity in both

**Table 1** Summary of smart surface with switchable wettability

| Responsive materials | Stimuli | Reversible wetting performances | Features in surface control | Advantages | Disadvantages | References |
|---|---|---|---|---|---|---|
| Thermosensitive polymer | Temperature | Hydrophilicity and underwater superoleophobicity /hydrophobicity and superoleophilicity transition | Surface composition | Facile operation, without chemical addition | Longer responsive time | [49] |
| Inorganic semiconductor oxides, organic light-sensitive polymers | Light | Hydrophobicity and superhydrophilicity transition | Surface composition | Rapid, facile operation, remote control | Specific wavelength | [29, 61] |
| Conductive polymers or substrate | Electric | Hydrophilicity and superhydrophobicity transition | Surface energy | Super-fast responsiveness, convenient controlment | Low safety factor, complex fabrication | [70, 73, 77, 78] |
| Magnetic particles/fluids | Magnetic | Dynamic wetting transition | Surface microstructure | Safety, fast responsiveness, easy to operate, low energy consumption, eco-friendly | Magnetic material assistance | [87–89] |
| SMPs, flexible material | Mechanical stress | Dynamic wetting transition | Surface microstructure | Fast responsiveness, continuous, eco-friendly | Contact reaction | [93–95] |
| Pyridine, carboxyl, and tertiary amine groups | pH | Superoleophilicity and hydrophilicity/ superoleophobicity transition | Chemical composition | Easy operation, wide application | Long responsive time, low sensitivity, heavy metal or complex preparation | [99, 102, 103] |
| Metal-ion, amine or nitrogen heterocycles with quaternized moiety | Ion | Superhydrophilicity and hydrophobicity transition | Chemical composition | Easy to operate | Specific reaction | [114, 117] |
| Surrounding media | Solvent | Superhydrophilicity and hydrophobicity transition | Interfacial free energy | Easy to operate | Only sensitive to one or a few specific solvents | [126] |

surface microstructure and chemistry for the first time (Fig. 10b). Moreover, the authors designed a rewritable platform for repeatedly creating different gradient wettings with four parts, *i.e.* upright pillars with hydrophobicity, collapsed pillars with hydrophobicity, collapsed pillars with hydrophilicity and upright pillars with hydrophilicity. Based on this route, it was demonstrated that the surface possessed an excellently tunable wettability, which may expand to other wetting-dependent functional applications.

## 4 Applications of smart wettability

Recently, smart materials with special controllable wetting surfaces have attracted an increasingly attention in many fields. Especially, lots of relevant researches mostly have centered upon oil/water separation, programmable transportation, anti-biofouling, detection and delivery, smart soft robotic and others.

### 4.1 Oil-water separation

Oil-water separation is a hot topic in the industry field and daily life due to the oil leakage, increasing oily industrial wastewater and so on[108,134,135]. Recently, materials with special interfacial superwettability have proven to be highly efficient to solve the practical problem, such as membrane-based separation materials, porous sponge-based absorption materials, metal/covalent-organic framework meshes, porous carbon materials, fabrics, and coatings *etc*[136–140]. Comparatively, the smart materials with switchable wettability by external excitation also can be used for complex environments and mixed solvents with high flux and high selectivity, which is favor to separate the oil/water mixtures on-demand, efficiently and energy-savingly[141–143].

The reversible wettability between superhydrophobicity/underwater superlipophilicity and superhydrophilicity/underwater superoleophobicity has received extensive attention. The smart surface with switchable wettability selectively presents two processes between "oil-removing" and "water-removing"[144]. While the surface is superhydrophobic/superoleophilic in air, the

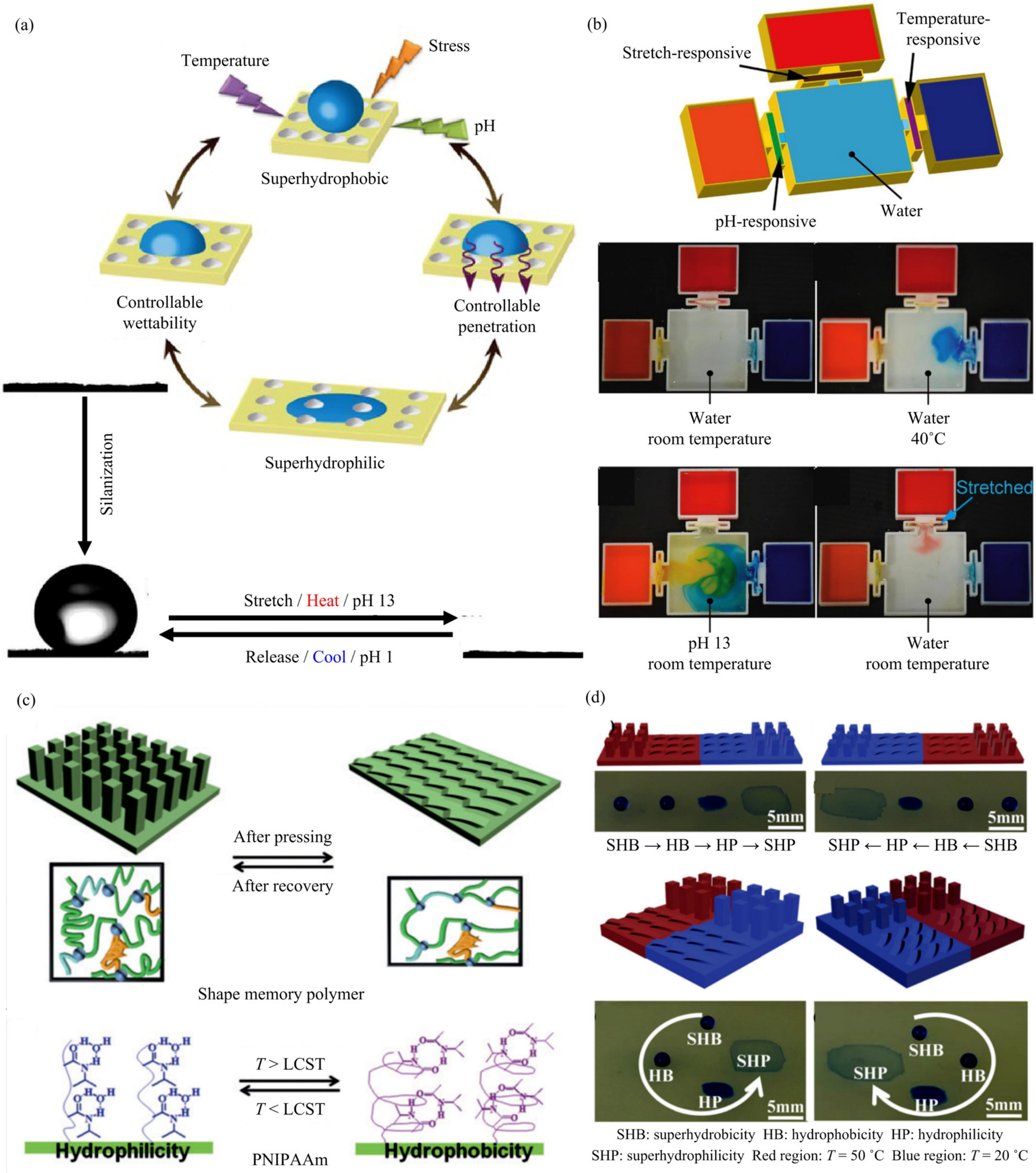

**Fig. 10** (a) Controlling the wettability by the stress/temperature/pH-responsive composite material, and presenting a multiple responsive system and wetting changes by the outside contain dyes of different colors. While stretched the stretch-responsive material, the red dye will diffuse into the center. Similarly, the 40 ˚C water induced the blue dye diffusing into the center, and a basic solution of pH 13 aroused the orange dye and blue dye diffusing into the center due to the exertion of stimuli[132]. (b) Surface fabrication by combining shape memory effect of SMPs and temperature-responsive PNIPAAm, applying as a rewritable platform for creating different gradient wettings by controlling surface microstructure and chemistry simultaneously[133].

heavy oil can pass through the film while retaining the water, working in an oil removing mode. While the surface is superhydrophilic/underwater superoleophobic, the water could spontaneously permeate through while preventing light oil from passing, belonging to the water removing mode. Based on the pH-responsive materials of thiol molecules, smart copper foams with reversible wettability between superhydrophobicity and hydrophilicity were used for the separation of oil/water mixtures with high efficiency in the neutral solution or basic solution bidirectionally in our previous work[105]. As shown in Fig. 11, while the pH increased, the surface wettability transformed into superhydrophilicity and underwater superoleophobicity, the "water-removing" mode allowed the water in the oil-water mixture to pass through the foam and into the beaker below. Nevertheless, while the pH decreased, as for the mixtures of dichloroethane and water, there appeared a opposite separation phenomenon of oil permeability.

### 4.2 Programmable transportation

In addition to the control of static wetting, the surface wettability transition also exhibits in the dynamic wettability by the droplet sliding property. Programmable droplet transportation is usually caused by changes in dynamic wettability under external stimuli, showing the difference in contact angle and anisotropic wetting behavior in two directions, which can be applied in various fields, such as biomedicine for non-powered micro-drug delivery, controllable self-lubricating transfer in mechanical engineering, controllable microfluidics system and long-distance water delivery for agricultural drip irrigation *etc*[145–148]. Inspired by the microstructure of stomata distributed on plants, Zhao *et al*. prepared a surface with programmable wettability arrays by using microfluidic emulsification templates, achieving the printing with mask integration because of the property of controllable droplet sliding on programmable wettability pathways and effective droplet transfer (Fig. 12b)[147]. Zhang *et al*. fabricated a temperature-adaptive V-shaped Prism Microstructures (VPM) surface to realize on-demand switchable direction transport of liquid *in situ* and real-time upon microarrays and thermal stimuli, which was unidirectional, bidirectional and directionally switchable transportation, so as to achieve precise liquid transport according to the path of the microfluidic channel (Fig. 12c)[149]. Inspired by the lotus and pitcher plants, Li and his teamwork fabricated a PDMS@$Fe_3O_4$ fabric surface *via* one-pot method, realizing a switchable surface between the superhydrophobic state and slippery state under external magnetic field, which can achieve anisotropic water transmission and programmable fog harvesting[150].

### 4.3 Anti-biofouling

The phenomenon of biological adhesion and aggregation on the material surface has brought great harm to the actual life and production of human beings, which has caused a great impact on the medical, food, shipping and others[151–153]. The biofouling processes usually contain non-specific adsorption of biomolecules (lipids,

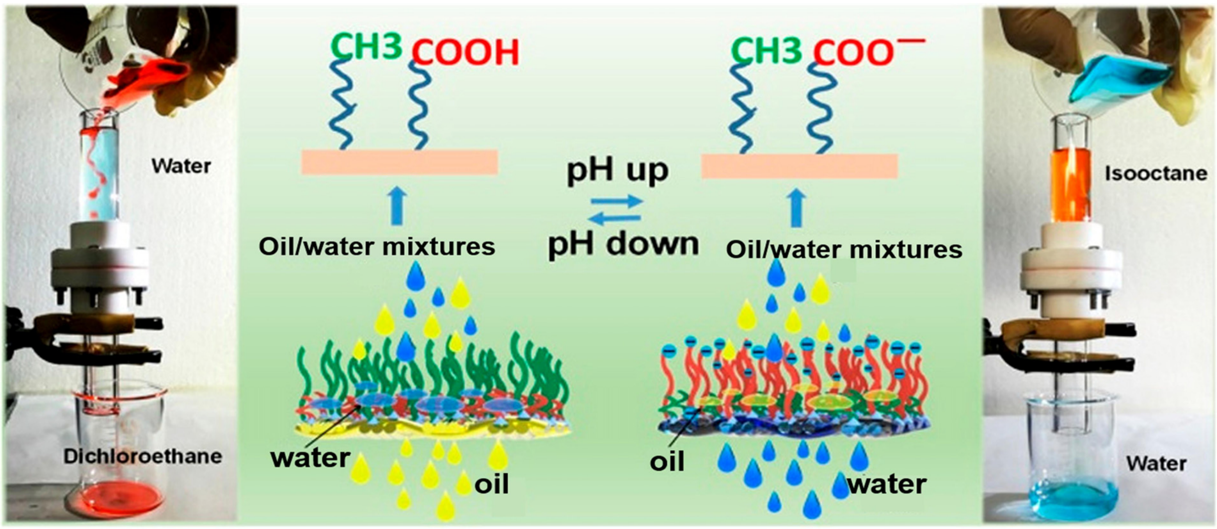

**Fig. 11** Controllable oil/water separation mode for oil-water mixtures with different pH values. Left, oil-removing mode, while pH up, water passed through the foam, whereas oil remained in the upper glass tube; Right, water-removing mode, while pH down, oil passed through the foam while water remained on the top[101].

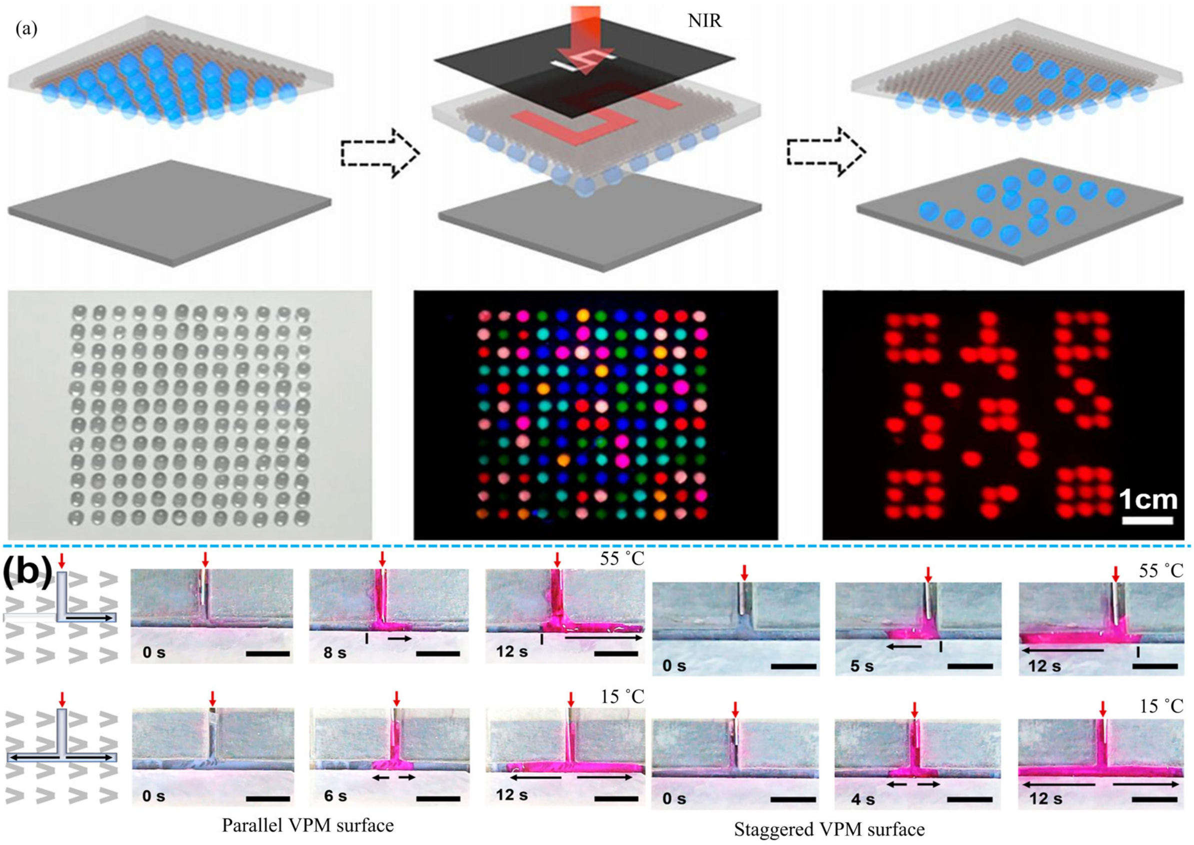

**Fig. 12** (a) Schematic diagram of patterned droplets' release with photomask and droplet array of hidden 2D code information[147]. (b) On-demand directional liquid transport in microfluidic channels[149].

glycans, proteins, *etc*.), molecular recognition-regulated cellular behaviors, and the formation of bacterium biofilms. According to this characteristic, smart materials interface with switchable wettability plays an important role in controlling the capture and release of cells, bacteria, and proteins[154–157]. Hou and coworkers developed a nanomaterial platform with controlling the capture and release of Circulating Tumor Cells (CTCs) by switching wettability between hydrophobic (at 37 ˚C) and hydrophilic (at 4 ˚C) upon grafting PNIPAAm onto surface (Figs. 13a and 13b)[158]. Based on the thermo-responsive polymers, Li *et al*. obtained a smart surface for adsorbing or desorbing fibronectin *via* electrospinning technology, which is due to the surface wettability transformation caused by the structural changes under different temperature[159].

Meanwhile, the smart wettability surfaces with capability of killing and releasing bacteria offer a new methodology for the practical applications in the biomedical and biotechnology fields. Yang *et al*.[160] presented a facile method for PNIPAAm functional surfaces by *in situ* preparation of silver nanoparticles (Ag NPs), which offered a "smart" antibacterial capability by controlling the surface wettability upon response to the change of environmental temperature. Large numbers of *E. coli* were killed at 37 ˚C and released at 4 ˚C on demand (Figs. 13c and 13d). Fu *et al*. fabricated smart antibacterial fabrics with the pH-responsive switchable wettability *via* the cross-linking reaction of poly(ureaformaldehyde) (PUF) NPs contained methylol and hexamethylene diisocyanate[161]. Since the acid solution will affect the $N^+$ concentration on the surface, the surface wettability transformed rapidly under different pH solution. Due to the transition of surface wettability from superhydrophobicity to superhydrophilicity, the absorption rate of bacteria changed, leading to the change of bactericidal rate accordingly. As for the bactericidal rate, the values were 80.0% and 83.33% corresponding to solution of pH = 7 and pH = 13, whereas the sterilization rate slightly increased to

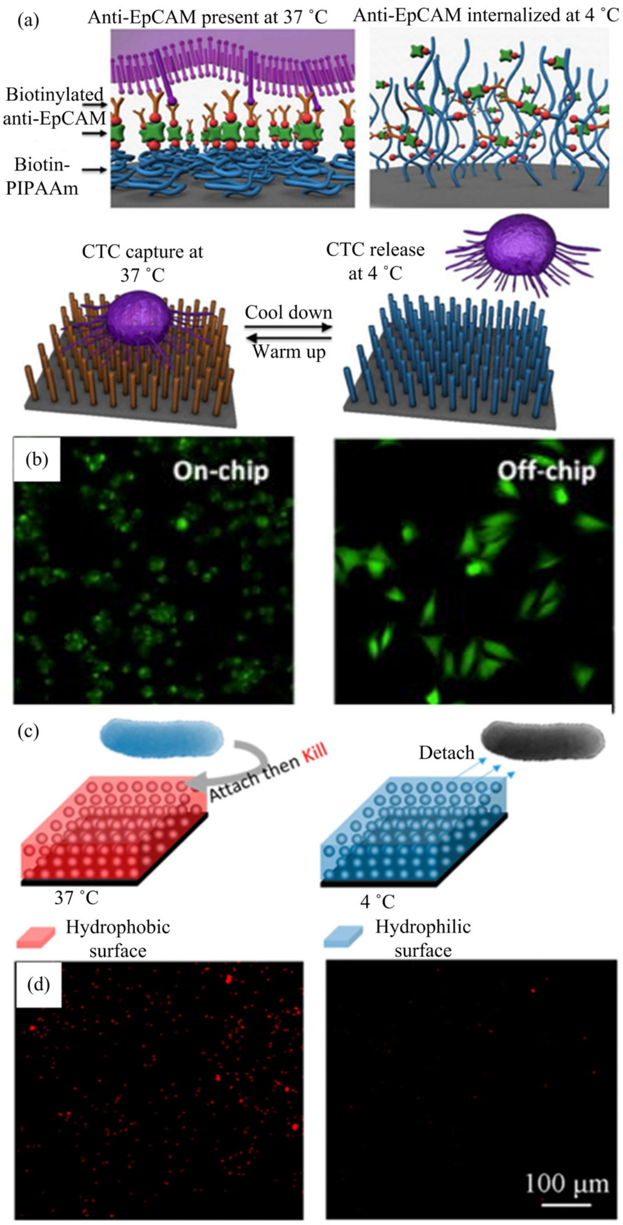

**Fig. 13** (a) Conceptual illustration of a nanomaterial-based platform for cell-affinity assay capable of capturing CTCs with superb efficiency and releasing upon stimulation these captured CTCs under reduced temperature, and (b) the successful culture of CTCs after capture and released from prepared specimen[158]. (c) Schematic of bacterial attachment and detachment in response to temperature variation, and (d) the corresponding confocal microscope fluorescence graphs of *E. coli*[160].

88.67% under pH = 1.

## 4.4 Detection and delivery

The smart wettability surfaces also have applied in drug transportation and detection, which provides a new way for the development of convenient and fast detection platforms and new drugs[162,163]. Liu *et al*. fabricated a Flexible Drug Release Device (FDRD) powered by a triboelectric nanogenerator (TENG), realizing the collection of biomechanical energy and converting it into electrical energy[164]. Induced by the electric effects by the on/off of mechanical switch, the wettability of the poly(3-hexylthiophene) P3HT layer in $Na_2SO_4$ aqueous solutions changed from hydrophobicity to hydrophilicity, so as to release the small molecules, such as methylene blue, fluorescein sodium and rhodamine 6 G from the FDRD, realizing the controllable and continuable release of drug. Different from the traditional detecting techniques, visual assay in detection have attracted lots of researchers' attention, which is mainly dependent on the changes of wetting behaviors under stimuli, including the static and dynamic state[165]. In virtue of rapid pH-responsive switchable superwettability between superhydrophilic and superhydrophobic, Jiang's team implemented a platform of naked-eye point-of-care testing for detecting pH, glucose and urea sensitively and visually just by observing the droplets state on the surface, so as to obtain the non-invasive diagnosis of diabetes for quantitative biosensing (Fig. 14)[166].

Moreover, to meet the Sustainable Development Goals of United Nations (Zero Hunger, Clean Water and Sanitation), it is necessary to improve the ability of crops to resist the climate and increase the utilization rate of fertilizers and pesticide[167,168]. Gao *et al*. fabricated a switchable Light-Responsive Herbicide Nanosafener (LHNS) by using $TiO_2$, biochar (BC) and hydrogen silicone oil, which provided a promising method for reducing the adverse effects of herbicides and improving the efficiency of foliar fertilizer utilization (Fig 15a)[169]. Adjusted by ultraviolet light, the hydrophobic surface changed into hydrophilic surface because of the presence of $TiO_2$, thereby increasing the adhesion of urea and promoting the growth of crops. Under infrared (IR) light, the photothermal effect of BC decreased the water absorption, so that the hydrophobic surface can be used as a glycine safener. In order to quickly and accurately detect the trace level picloram (PCR) in agriculture and environment, Mutharani *et al*. adopted poly(N-vinylcaprolactam) and multi-walled carbon nanotubes (MWCNT-PVCL) onto the electrochemical sensors with gold nanoparticles (Fig. 15b)[170]. Using

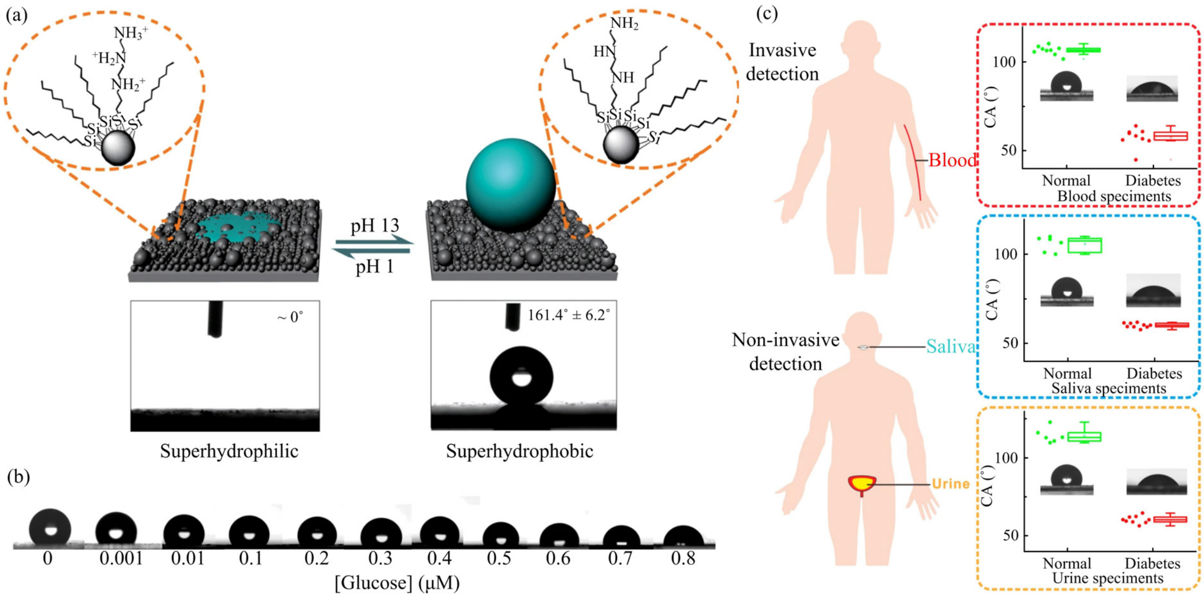

**Fig. 14** (a) Working principle of the switching of the pH-responsive superwetting surface properties. (b) Changes of wetting states of droplets with different concentrations of urea or glucose. (c) Visual quantitative detection of glucose in practical settings[166].

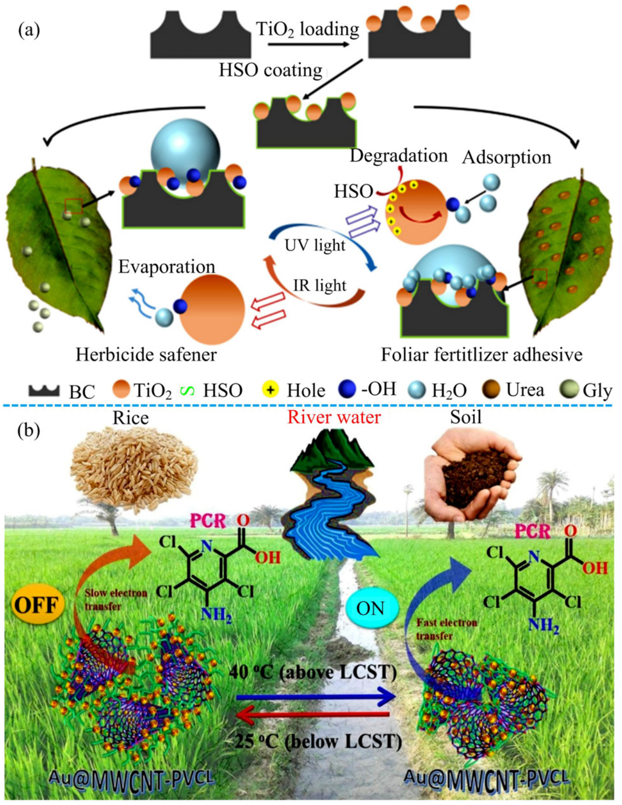

**Fig. 15** (a) The light-responsive smart surface used for herbicide safener or foliar fertilizer adhesive, hydrophilic LHNS can improved the adhesion of foliar fertilizer; hydrophobic LHNS can prevent the adsorption of the amount of water and used as a glycine safener[169]. (b) The smart surface upon temperature for PCR detection, the hydrophilic surface of PVCL at 25 ˚C prevent the PCR pass over in "OFF" state, while the switched hydrophobic surface at 40 ˚C upgrade PCR oxidation in "ON" state[170].

the temperature-sensitive catalyst characteristics, it can be used to realize the conversion of surface wettability and affect the electrocatalytic active sites, so as to realize the controllable and selective detection of PCR, with a 1.5 nM limitation of detection for PCR at 40 ˚C.

### 4.5 Smart soft robotic

As an emerging field, soft robotics combines traditional robotics with material science and chemistry to improve performance by implementing novel hardware concepts and functions[171]. As mentioned in above, the control of switchable surface wettability mostly is caused by the properties a wide range of material. Inspired by multimodal locomotive strategies of insects, Chen *et al*. reported a hybrid terrestrial-aquatic robot by using electrowetting to control surface tension magnitude, realizing the transition between water surface and underwater[172]. With the effects of electrical signal on the all four EWPs, the microrobot made use of the combination of surface tension and buoyancy to achieve controllable sinking (Fig. 16a). Moreover, the microrobot could overcome surface tension and climb a modest incline to transition back onto land, exhibiting unique locomotive capabilities (Fig. 16b).

Usually, such as the capability of droplet capture, storage, release, and pumpless transportation by the

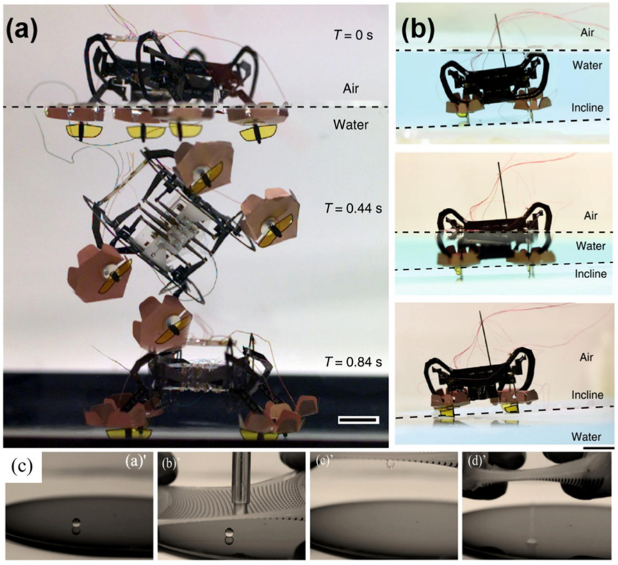

**Fig. 16** (a) Images of a robot sinking into water with a 600 V signal on the all four EWPs[172]. Scale bars is 5 mm. (b) The robot climbs an incline from fully submerged in water, the air-water interface, to completely exiting water[172]. Scale bar is 1 cm. (c) Droplet pick-and-place with the composite surface on a Si wafer[174].

reversible changes in surface adhesion can be used as a "mechanical hand" for no-loss droplet transfer[87,106,108,173]. A black silicon/elastomer composite surface with highly flexibility was designed by Park and coworkers, which presented switchable wettability as well as adhesion transformation between slippery lotus and adhesive rose petal states under various mechanical strains[174]. Moreover, due to this characteristic, the self-propulsion and pick-and-place of water droplets were obtained, which was expected to be used in microfluidic field (Fig. 16c). Wang *et al*. introduced a mechano-regulated device to capture, transport and release oil droplets by moving microfiber array, exhibiting the water-droplet-capturing surface of dandelion seeds and water-droplet-repelling surfaces of lotus leaves[175].

In addition, the smart surface with switchable wettability can not only control the adhesion of droplets but also the gas and underwater oil, further helping to provide new opportunities for the development of smart switchable surfaces. Guo's team produced a smart superwetting surface with the properties of manipulation oil droplets and bubbles underwater *via* switchable adhesion upon ultraviolet response[176]. Liu *et al*. reported a novel oil-triggered surface by switching wettability property between lotus-leaf and nepenthes, which was used in various droplet manipulation modes with real-time property[121].

Last but not least, smart wettable surfaces are also used in other fields, condensation heat transfer[177], self-cleaning prints[178], smart textile[179], encryption[180] and so on. There is no doubt that smart surfaces with switchable wettability will bring progress to our lives, and provide new ideas and approaches for the development of industrial sets, biomedical materials and smart agricultural materials.

## 5 Conclusions and outlook

In this review, we have reviewed the several interfacial superwetting states based on the surface of natural creatures with special microstructures, including special wetting models and liquid adhesive behaviors, so as to provide wetting mechanisms for switchable wettability on superwettable materials. At the same time, we focus on the recent advances in different smart surfaces in response to external stimuli, mainly including physical stimuli (such as temperature, light, electric field, magnetic, stress), chemical stimuli (like pH, ions and solvents), and dual or multiple stimuli. By analyzing the responsive mechanism under different stimulus, we can find that the surface wettability transition is mainly caused by the transformation of the surface chemical composition, surface energy or surface microstructures. Simultaneously, with the introduction of the responsive materials, the surface wettability is controllable, effectively expanding the surface functional properties, so that these surfaces can be applied in oil/water separation, liquid manipulation, anti-biofouling, detection and delivery fields *etc*.

In summary, the smart wettability surfaces have made several progresses in recent years, but there still remain many problems. Firstly, the use of responsive materials should be environmentally friendly. Secondly, most of the surface responsiveness is single stimuli, which greatly limits its application fields. The complexity of the factual environment determines the surface to have multiple response characteristics. Aiming at meeting diverse requirements in different application environments, diverse smart surfaces should be further exploited. Thirdly, high sensitivity, fast response, repeatable response times, and responsive range are very critical. The electrical response speed is relatively fast, but the responsive process such as light and temperature requires a relatively long time. At the same time, there are problems with narrow response range, such as smaller response temperature span, smaller pH response range, broader responsive optical wavelength, etc. Fourthly, complex preparation procedures greatly limit the possibility of its application in production, so it is currently limited to laboratory research. In order to solve the aforementioned problems, the construction of smart surfaces should be emphasized on the environmentally-friendly materials, low cost, facile preparation process. Recently, smart materials provide a wider range of materials for 3D printing technology and expand the concept of 4D printing, which will be more conducive to the development of smart surfaces in the future. Last but not least, the mechanical and chemical long-term stability is critical in determining whether the smart surfaces can be successfully used in the actual applications. Therefore, how to bind the responsive materials onto the substrate tightly should also be a major concern in the future. Nevertheless, there will also emerge lots of opportunities in the field of intelligent transformation of bioinspired superwettability, including new bio-inspirations, coupled strategies, versatility and novel applications.

## Acknowledgment

The authors thank the National Natural Science Foundation of China (No. 51775231), National Postdoctoral Program for Innovative Talents (BX20180123), China Postdoctoral Science Foundation (2018M641782), Scientific Research Project of Jilin Provincial Department of Education (JJKH20211117KJ) and JLU Science and Technology Innovative Research Team (No. 2017TD-04).

**Open Access** This article is licensed under a Creative Commons Attribution 4.0 International License, which permits use, sharing, adaptation, distribution and reproduction in any medium or format, as long as you give appropriate credit to the original author(s) and the source, provide a link to the Creative Commons licence, and indicate if changes were made.

The images or other third party material in this article are included in the article's Creative Commons licence, unless indicated otherwise in a credit line to the material. If material is not included in the article's Creative Commons licence and your intended use is not permitted by statutory regulation or exceeds the permitted use, you will need to obtain permission directly from the copyright holder.

To view a copy of this licence, visit http://creativecommons.org/licenses/by/4.0/.
